# Oxide Two‐Dimensional Electron Gas with High Mobility at Room‐Temperature

**DOI:** 10.1002/advs.202105652

**Published:** 2022-02-20

**Authors:** Kitae Eom, Hanjong Paik, Jinsol Seo, Neil Campbell, Evgeny Y. Tsymbal, Sang Ho Oh, Mark S. Rzchowski, Darrell G. Schlom, Chang‐Beom Eom

**Affiliations:** ^1^ Department of Materials Science and Engineering University of Wisconsin‐Madison Madison WI 53706 USA; ^2^ Department of Material Science and Engineering Cornell University Ithaca NY 14853 USA; ^3^ Platform for the Accelerated Realization, Analysis, and Discovery of Interface Materials (PARADIM) Cornell University Ithaca NY 14853 USA; ^4^ Department of Energy Science Sungkyunkwan University (SKKU) Suwon 16419 Republic of Korea; ^5^ Department of Physics University of Wisconsin Madison WI 53706 USA; ^6^ Department of Physics and Astronomy University of Nebraska Lincoln NE 68588 USA; ^7^ Department of Material Science and Engineering Cornell University Ithaca NY 14853 USA; ^8^ Kavli Institute at Cornell for Nanoscale Science Ithaca NY 14850 USA; ^9^ Leibniz‐Institut für Kristallzüchtung Berlin 12489 Germany

**Keywords:** 2‐dimensional electron gas, room temperature high mobility, transparent conducting oxide, alkaline‐earth stannate

## Abstract

The prospect of 2‐dimensional electron gases (2DEGs) possessing high mobility at room temperature in wide‐bandgap perovskite stannates is enticing for oxide electronics, particularly to realize transparent and high‐electron mobility transistors. Nonetheless only a small number of studies to date report 2DEGs in BaSnO_3_‐based heterostructures. Here, 2DEG formation at the LaScO_3_/BaSnO_3_ (LSO/BSO) interface with a room‐temperature mobility of 60 cm^2^ V^−1^ s^−1^ at a carrier concentration of 1.7 × 10^13^ cm^–2^ is reported. This is an order of magnitude higher mobility at room temperature than achieved in SrTiO_3_‐based 2DEGs. This is achieved by combining a thick BSO buffer layer with an ex situ high‐temperature treatment, which not only reduces the dislocation density but also produces a SnO_2_‐terminated atomically flat surface, followed by the growth of an overlying BSO/LSO interface. Using weak beam dark‐field transmission electron microscopy imaging and in‐line electron holography technique, a reduction of the threading dislocation density is revealed, and direct evidence for the spatial confinement of a 2DEG at the BSO/LSO interface is provided. This work opens a new pathway to explore the exciting physics of stannate‐based 2DEGs at application‐relevant temperatures for oxide nanoelectronics.

## Graphical abstract

The promise of BaSnO_3_ (BSO) band structure and scattering channels to relieve low room temperature mobility challenges of interfacial oxide two‐dimensional electron gases has been blocked by structural and point defects. The authors overcome this with a new synthesis approach combining a BSO atomically flat pseudo‐substrate layer with a band‐aligned BSO/LaScO_3_ oxide interface.

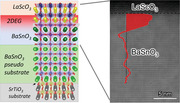

## 1 Introduction

Two dimensional electron gases (2DEGs) at oxide interfaces have attracted significant attention in both fundamental research and potential device applications. Among them, the heterointerface between LaAlO_3_ (LAO) and SrTiO_3_ (STO) is the most studied prototype system. Fascinating physical phenomena including magnetism,^[^ ^1^ ^]^ superconductivity,^[^ ^2^, ^3^ ^]^ strong spin–orbit interactions,^[^ ^4^, ^5^ ^]^ and exotic quantized transport^[^ ^6^, ^7^ ^]^ have been reported. Unfortunately, despite extensive work on STO‐based 2DEG heterostructures with overlayers such as LaAlO_3_,^[^ ^8^ ^]^ LaTiO_3_,^[^ ^9^ ^]^ NdGaO_3_^[^ ^10^ ^]^ and *γ*‐Al_2_O_3_,^[^ ^11^ ^]^ room‐temperature mobilities of interfacial 2DEGs are poor (e.g., < 10 cm^2^ V^−1^ s^−1^). This arises from the nature of electron states in the narrow Ti *d*‐bands that host the 2DEG in STO, their interaction with the crystalline lattice, and multiple interband scattering channels due to the degenerate *t*_2g_ orbital symmetry.^[^ ^12^, ^13^ ^]^ This has stymied wide‐ranging room‐temperature 2DEG applications in these systems.

One route toward higher room‐temperature interfacial 2DEG mobilities is to move away from STO to a non‐polar oxide with more dispersive *s* or *p* orbital‐based conduction bands to provide highly mobile carriers at room temperature. BaSnO_3_ (BSO) has gained significant attention in theory and experiment as an alternative interfacial 2DEG host material.^[^ ^13^, ^14^, ^15^ ^]^ The conduction band of BSO is composed of highly dispersive non‐degenerate *s*‐orbitals with a large band width and a low effective mass. Additionally, the interband scattering channel can be turned off from the isotropic *s* orbitals like conduction band structure, resulting in a longer lifetime for the charge carrier.^[^ ^13^ ^]^ Therefore, the BSO based 2DEG's mobility at room temperature is predicted to be two orders of magnitude higher than that of STO based 2DEGs in a structurally perfect BSO host.^[^ ^16^, ^17^, ^18^ ^]^

The sensitivity of carrier mobility and free carrier concentration to structural and point defects makes achieving a high mobility 2DEG in BSO challenging. One such structural defect is the high density of dislocations in typical BSO thin films (more than 10^9^ cm^–2^).^[^ ^19^, ^20^, ^21^, ^22^ ^]^ These threading dislocations originate from the large lattice mismatch between BSO and commercially available perovskite substrates (ranging from −5.2% (SrTiO_3_) to −2.4% (PrScO_3_)). Because of such defects, the mobility of electrons produced by La doped thin films of BaSnO_3_^[^ ^19^ ^]^ has not yet exceeded that of La‐doped BaSnO_3_ bulk single crystals.^[^ ^16^, ^17^ ^]^ Another compromising factor is the formation of complex point defects during BSO film growth, which act as additional electron traps or scattering sites.^[^ ^19^ ^]^ Finally, producing an interfacial 2DEG in a BSO‐based bilayer heterostructure requires proper band structure alignment and a good epitaxial match between the top oxide layer and BSO.

For these and other reasons, only a few experimental studies have pursued BSO‐based 2DEG formation, using either modulation doping or polarization doping.^[^ ^23^, ^24^, ^25^, ^26^ ^]^ A recent report has claimed an interfacial conductivity improvement at the LaInO_3_/La‐BaSnO_3_ interface, akin to the polar catastrophe polarization doping scenario.^[^ ^23^, ^24^, ^25^ ^]^ La‐doped (0.3%) BSO channels were, however, used to compensate a high density of defect states at the LaInO_3_/BaSnO_3_ interface. Prakash et al. reported a modulation doping approach utilizing La‐SrSnO_3_/BaSnO_3_ heterostructures, where electrons from the La‐doped SrSnO_3_ side spill over into an undoped BSO layer.^[^ ^26^ ^]^ They found that the modulated electrons spread over 3–4 unit cells toward the BSO layer, even though La‐doped SrSnO_3_ layer is the more dominant conducting path than the modulated BSO layer. Nonetheless, the reported 2DEG's carrier behaviors did not unambiguously demonstrate 2DEG confinement at the heterointerface.

**Figure 1:**
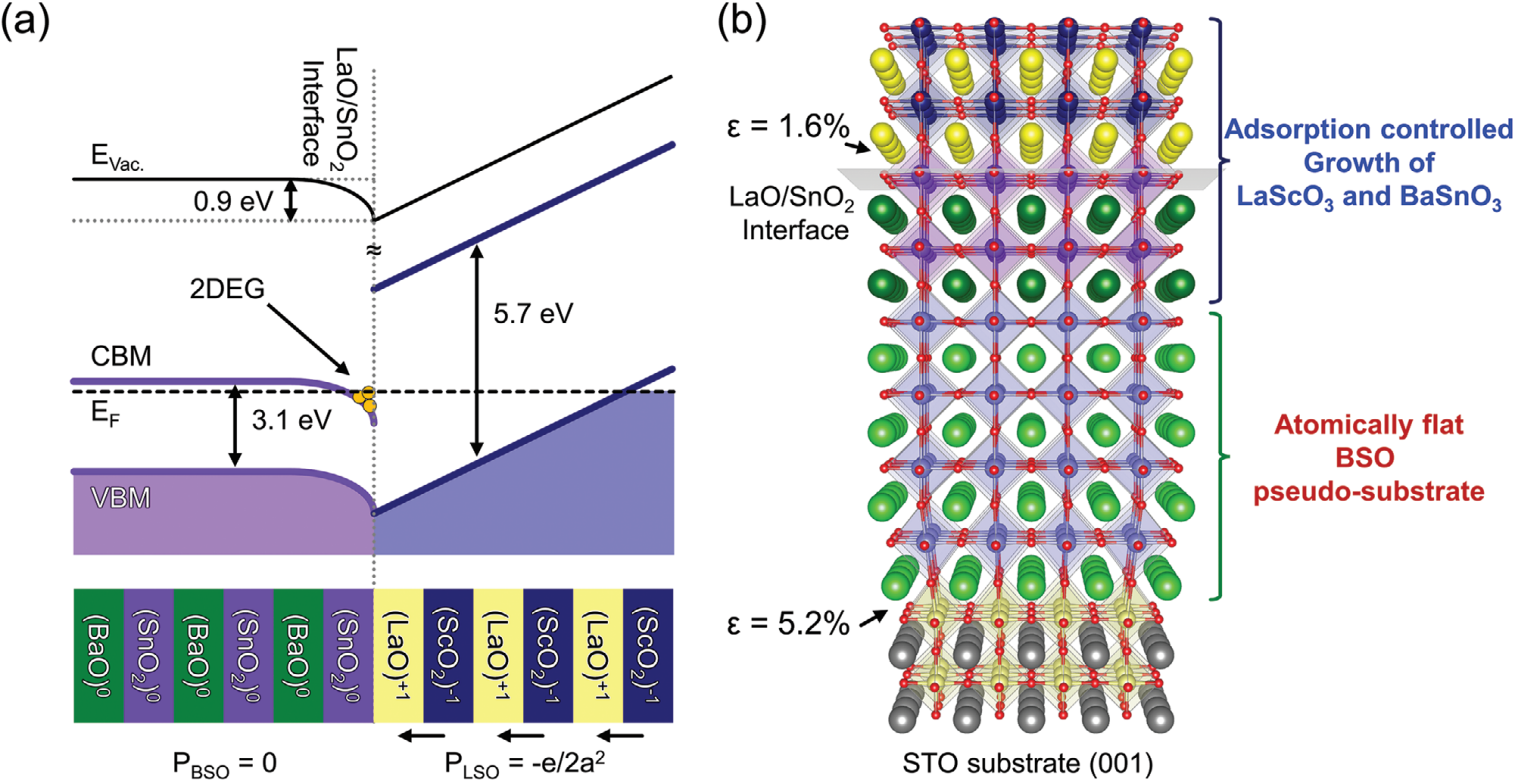
Experimental design for 2DEG formation at the LSO/BSO interface. a) The band diagram of LSO/BSO heterostructures above the critical thickness, showing 2DEG formation at the interface. b) Schematic of a BSO pseudo‐substrate (550 nm thick BSO film on STO (001) substrate) and our strategy to acquire high quality 2DEGs at the LSO/BSO interface. This process minimizes the dislocation scattering centers in the MBE‐grown BSO 2DEG channel layer on an atomically flat SnO_2_‐terminated annealed BSO pseudo‐substrate. The annealing treatment reduces the dislocation density and produces SnO_2_‐terminated atomically flat surfaces. The structure consists of MBE‐grown LSO (several unit cells) on top of the adsorption‐controlled BSO thin film (45 nm) that is also grown by MBE on the BSO pseudo‐substrate.

Here we report a BSO‐based highly mobile interfacial 2DEG, where we have overcome the electronic alignment and defect density issues discussed above. We incorporate LaScO_3_ (LSO) as a top polar layer epitaxially registered with a non‐polar host BSO thin film. LSO is orthorhombic, but we give pseudocubic LSO thicknesses throughout this manuscript. Similar to the case of LAO/STO, the LSO/BSO interface has a band alignment that facilitates 2DEG formation^[^ ^13^ ^]^ (see **Figure** **1**a, LSO/BSO band diagram) while possessing excellent structural coherency. The mismatch strain is less than 1.6%, as is evident in the atomic model of the LSO/BSO interface shown in Figure 1b. The LSO/BSO interface hosts a polar discontinuity (Figure 1a),^[^ ^13^ ^]^ facilitating 2DEG formation at atomically sharp LaO/SnO_2_ interfaces. We dramatically reduced the BSO defect densities by first growing a thick BSO layer by pulsed‐laser deposition (PLD), annealed it at high temperature ex situ, and then continued the growth of BSO by MBE to form the desired interface between BSO and the polar LSO layer. Electronic transport measurements revealed an insulator‐to‐metal transition at a threshold thickness (*t*_c_) of about 4 unit cells (u.c.), consistent with the polar catastrophe model,^[^ ^13^ ^]^ and a room‐temperature mobility as high as 60 cm^2^ V^−1^ s^−1^ with a 1.7 × 10^13^ cm^–2^ carrier density. In‐line electron holography showed negative charges confined to the LSO/BSO interface. TEM analysis verified a reduced dislocation density resulting from our synthesis approach. Complete structural and morphological analysis demonstrates high crystalline quality. This first demonstration of a high mobility BSO‐based 2DEG provides a fascinating platform for exploring transparent conducting oxide electronic devices and the physics of two‐dimensional *s*‐orbital systems.

## 2 Results and Discussion

**Figure 2:**
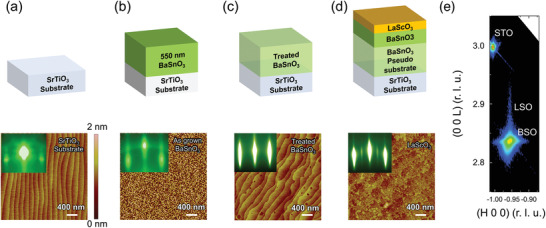
Fabrication of a high mobility 2DEG at the LSO/BSO interface, showing AFM and RHEED (bottom) after each step of the fabrication (top). a) STO (001) substrate, b) as‐grown 550 nm thick BSO film (BSO pseudo‐substrate), c) after water leaching and thermal annealing of BSO pseudo‐substrate, and d) LSO (10 u.c.)/BSO (45 nm) grown on the BSO pseudo‐substrate via MBE. The insets in the AFM images represent the RHEED patterns at each step of the PLD (steps (a) and (b)) and MBE growths (steps (c) and (d)). e) Reciprocal space mapping (RSM) around 103 reflections from LSO (10 u.c.)/BSO (45 nm) grown on the BSO pseudo‐substrate.

Our approach to minimizing dislocation density starts with a PLD‐grown thick BSO buffer layer (about 550 nm) (Experimental Section), as shown in **Figure** **2**. The thick BSO layer is leached in water for 15 s and then ex situ annealed in oxygen at 1150 °C for 2 h (which we refer to as the BSO pseudo‐substrate hereafter)^[^ ^27^ ^]^ before MBE regrowth. We note that undoped BSO buffer layers about half this thickness were used to achieve the highest La‐doped BaSnO_3_ single film electron mobilities to date.^[^ ^19^ ^]^ We summarize the structural analysis of the BSO pseudo‐substrate before and after thermal treatment in Figure S1 (Supporting Information). This treatment not only reduces the dislocation density,^[^ ^28^ ^]^ but also produces SnO_2_‐terminated atomically flat surfaces. The full width at half maximum (FWHM) of the BSO 002 peak's rocking curve is 0.013 degrees after treatment (Figure S1d, Supporting Information). The in‐plane and out‐of‐plane lattice constants of the BSO pseudo‐substrate obtained from the reciprocal space maps of 103 BSO peaks (Figure S1f, Supporting Information) are 4.112 and 4.118 Å, respectively, indicating an almost fully relaxed state.^[^ ^29^ ^]^ Atomic force microscope images show an atomically flat surface of the BSO pseudo‐substrate with single unit cell steps of 0.4 nm (Figure 2c).

After loading the BSO pseudo‐substrate into the MBE, we grew a 45 nm thick BSO layer in an adsorption‐controlled regime, followed by a 10 u.c. thick LSO layer (Figure 2d) (Experimental Section; Figure S2, Supporting Information). Using this method, we form the LSO/BSO interface away from the air‐exposured BSO surface, yet capitalize upon the benefits of the pseudo‐substrate with lowered threading dislocation density and desired SnO_2_‐terminated surface. Further, the 2DEG interface is produced in the MBE‐grown portion of the structure known to produce high‐mobility BSO layers. A reciprocal space map around the (103) STO substrate peak shows that the LSO film is fully coherent with respect to the underlying BSO film (Figure 2d).

**Figure 3:**
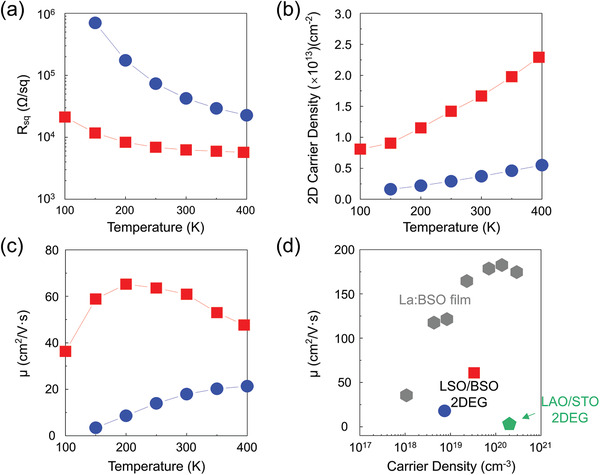
Transport properties of the LSO/BSO heterostructures. a) Sheet resistance, b) carrier density, and c) mobility of LSO (10 u.c.)/BSO (60 nm) directly grown on an STO (001) substrate (closed blue circle) and LSO (10 u.c.)/BSO (45 nm) grown on the BSO pseudo‐substrate (closed red square). d) Electron mobility at 300 K as a function of carrier density for 2DEGs at oxide heterointerfaces in this work, La:BSO film^[^ ^19^ ^]^ and LAO/STO^[^ ^30^ ^]^ reported in the literature.

**Figure** **3** shows the temperature‐dependent transport properties of the 10 u.c. thick MBE LSO/BSO 2DEGs grown on the BSO pseudo‐substrate (red squares). It is compared to a control sample of 10 u.c. LSO/BSO (60 nm) 2DEGs grown without the BSO pseudo‐substrate (directly grown on STO (001) substrate by MBE, blue circles). Both systems show semiconducting‐like features across the entire measurement temperature range between 100 and 400 K (Figure 3a). The carrier density of the LSO/BSO 2DEG grown on the BSO pseudo‐substrate decreases monotonically over the temperature range and shows a 4.5 times higher carrier density than the control sample (Figure 3b). In addition, the LSO/BSO 2DEG grown on the BSO pseudo‐substrate has 6.8 times lower sheet resistance and 3.4 times higher mobility (Figure 3c) than the control sample. Notably, the highest 2DEG mobility at room temperature is 60 cm^2^ V^−1^ s^−1^ with carrier concentration 1.7 × 10^13^ cm^–2^. This is an order of magnitude higher 2DEG mobility at room temperature than in STO‐based 2DEGs.^[^ ^8^, ^9^, ^10^, ^11^, ^30^ ^]^

**Figure 4:**
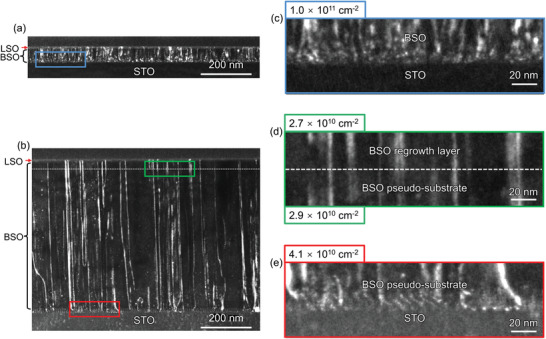
Threading dislocation density approximated from the cross‐sectional TEM weak beam dark‐field images. Weak beam dark‐field images of a) LSO (10 u.c.)/BSO (60 nm)/STO without a BSO pseudo‐substrate and b) LSO (10 u.c.)/BSO (45 nm) grown on the BSO pseudo‐substrate. c) Magnified image at the BSO/STO interfaces of LSO (10 u.c.)/BSO (60 nm)/STO. d) Interface between the BSO pseudo‐substrate and BSO regrowth layer, indicating little change in defect density. e) Magnified image at LSO (10 u.c.)/BSO (45 nm) grown on the BSO pseudo‐substrate and extracted dislocation densities showing reduced dislocation density as a result of high temperature annealing.

Our transmission electron microscopy (TEM) measurements quantify that the dislocation density of LSO/BSO heterostructures grown on the BSO pseudo‐substrate is reduced from the control sample, which underlies the observed enhanced room‐temperature mobility and carrier density. Misfit dislocations are known to be prevalent in BSO grown on STO substrate due to the large lattice mismatch. We found that threading dislocations propagate along the film growth direction (**Figure** **4**a,b) from the BSO/STO interface to the LSO layer (Figure S3, Supporting Information). Figure 4a shows the TEM weak beam dark‐field images of the control sample (10 u.c. LSO/BSO 60 nm grown on a STO (001) substrate). We evaluate a threading dislocation density on the order of ≈10^11^ cm^–2^ (Figure 4c) from the image analysis as shown in Figure S4 (Experimental Section; Supporting Information). The misfit dislocations at the BSO film‐STO substrate interface are denoted by red arrows (Figure S5b, Supporting Information). Notably, the LSO/BSO heterostructure grown on the BSO pseudo‐substrate showed a lower dislocation density of 4.1 × 10^10^ cm^–2^ (Figure 4e), less than half of the density observed when grown directly on an STO (001) substrate (Figure 4c). This is because the PLD grown BSO film was annealed at high temperature which has been shown to cause annihilation of threading dislocations.^[^ ^21^, ^28^ ^]^ The dislocation density of the BSO layer grown on the BSO pseudo‐substrate is very similar to that of the BSO pseudo‐substrate (Figure 4d). This provides less charge trapping and scattering, consistent with the increased carrier density and mobility at room temperature for the LSO/BSO heterostructure grown on the BSO pseudo‐substrate.

**Figure 5:**
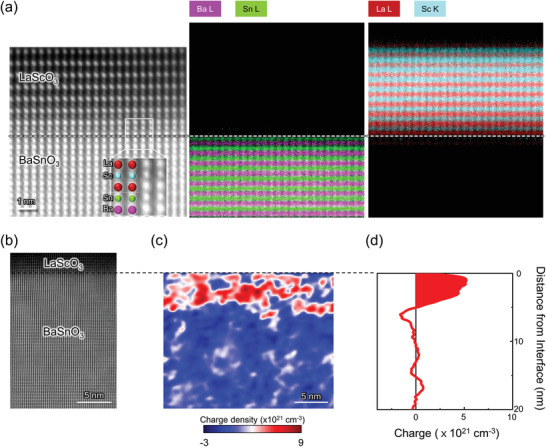
Elemental analysis and electron distribution at the LSO/BSO interface. a) Atomic resolution STEM‐HAADF images and EDS elemental mapping of the LSO/BSO interface, indicating an atomically abrupt interface. b) Direct imaging of the LSO/BSO interface by STEM HAADF imaging are shown next to c) the charge density maps and d) 1D electron density profiles obtained by in‐line electron holography for the LSO (10 u.c.)/BSO (90 nm) grown on an STO substrate.

We used in‐line electron holography to quantify 2DEG confinement near the interface, and to support the LSO thickness‐dependent electrical transport properties of the LSO/BSO interface (Experimental Section; Supporting Information). For these measurements we grew the LSO layer by PLD on an MBE‐grown 90 nm thick BSO layer grown on a STO (001) substrate (Supporting Information). All BSO surface were SnO_2_ terminated with single unit cell steps, achieved by a water leaching treatment.^[^ ^27^ ^]^ The LSO film thickness was controlled by reflection high‐energy electron diffraction (RHEED) intensity oscillations (Figure S6, Supporting Information). Atomically resolved STEM‐energy‐dispersive X‐ray spectroscopy (EDS) elemental mapping across the interface verified the LaO/SnO_2_ termination of the LSO/BSO interface (**Figure** **5**a) (Experimental Section and Supporting Information). Different LSO thickness heterostructures established a critical thickness (*t*_c_) of 4 unit cells for conductivity (Figure S7, Supporting Information), consistent with a polar catastrophe interpretation.^[^ ^31^ ^]^ In‐line electron holography results of a 4 u.c. thick LSO/BSO interface (Figure S8, Supporting Information) shows no significant net charge density near the interface, while those of the 10 u.c. LSO/BSO (Figure 5b) show a 2D charge density equivalent to 5 × 10^21^ cm^–3^, distributed with a peak 1.5 nm below the LSO/BSO interface before quickly decaying to zero around 5 nm below the interface. There is no long tail of electron density extending deep into the BSO side.

This depth dependence is in contrast that obtained from in‐line holography results of LAO/STO heterostructures, which show a charge confinement within 1.5 nm below the interface and a maximum electron density of 5 × 10^21^ cm^–3^ located 0.5 nm below the interface.^[^ ^32^ ^]^ We attribute these differences primarily to the lower dielectric constant of BSO compared to STO.^[^ ^33^, ^34^ ^]^ A secondary reason could be the difference between Sn 5*s* orbitals in BSO and Ti 3*d* orbitals in STO from which the 2DEG electron states arise. A related broadening of the 2DEG extent (4.5 nm) was reported in the (111) oriented LAO/STO 2DEGs.^[^ ^32^ ^]^ (111)‐oriented LSO/STO 2DEGs due to orbital orientation in STO. It is worth noting that the carrier density of LSO/BSO 2DEGs obtained by a Hall effect measurement is a much smaller value than that measured by inline holography. This discrepancy may come from the fact that the Hall effect measurement is sensitive to mobile charge carriers, whereas inline holography reflects the total charge density, including both mobile and localized charges.^[^ ^32^ ^]^

Our large room‐temperature mobility of 60 cm^2^ V^−1^ s^−1^ (Figure 3c) is still lower than that predicted theoretically.^[^ ^13^ ^]^ Interfacial cation intermixing at the LSO/BSO interface could reduce the carrier mobility, a common phenomenon in oxide interfaces. The STEM‐EDS mapping results shown in Figure 5a, however, do not indicate significant cation intermixing at the LSO/BSO interface. Our precise atomic‐column resolved STEM‐EDS composition profiles of the Sc‐K, Sn‐L, Ba‐L, and La‐L edge signals reveal that the atomic interdiffusion across the interface for both A site (La and Ba) and B site (Sc and Sn) is less than 1 nm (Figure S9, Supporting Information). This is similar to that observed in typical STO‐based 2DEG studies.^[^ ^35^ ^]^ Our in‐line holography results (Figure 5b) discussed above indicate that most of the carriers do not reside at the cation intermixed region of the BSO, but deeper into the BSO layer. This suggests that intermixing does not dominantly control LSO/BSO 2DEG mobility degradation.

Our LSO/BSO interfacial 2DEG mobility is also below that of La‐doped BSO films with similar measured dislocation densities (Figure 4) and similar mobile carrier concentration (Figure 3d).^[^ ^19^ ^]^ This indicates that dislocation density alone does not completely determine our 2DEG mobilities. For instance, dislocation cores are known to have abundant dangling bonds which effectively scatter electrons in semiconductor films.^[^ ^36^ ^]^ In fact, comparative STEM studies for both La‐doped BSO and undoped BSO films revealed distinctly different local atomic arrangements around dislocation cores.^[^ ^37^ ^]^ La ions in La‐doped BSO can accumulate inside dislocation cores, forming anti‐site defects (La_Sn_^–1^). This will screen the potential attributed to positive charges of the core oxygen vacancies known to reside at dislocation core in perovskite oxides,^[^ ^38^, ^39^, ^40^ ^]^ reducing the Coulomb scattering of conduction electrons. This indicates that the defect scattering by dislocation cores may be less in La‐doped BSO than in our undoped BSO.^[^ ^41^, ^42^ ^]^ We conclude that direct comparison of the dislocation density between La‐doped BSO films and LSO/BSO interfacial 2DEGs likely does not completely determine the observed differences in electron mobilities, although we have clearly shown that our improved growth technique reduces dislocation density and increases mobility in BSO interfacial 2DEGs. Lattice‐matched single crystal substrates^[^ ^43^, ^44^, ^45^ ^]^ should reduce the dislocation density even further, and are a promising path to achieving the highest mobility in BSO 2DEGs.

## 3 Conclusions

In conclusion, we have demonstrated a highly mobile 2DEG at the LSO/BSO interface with room‐temperature mobilities as high as 60 cm^2^ V^−1^ s^−1^. Future work will reveal whether the 2DEGs at the LSO/BSO interface can show the exotic interface physics, such as superconductivity,^[^ ^2^, ^3^ ^]^ a two‐dimensional hole gas (2DHG),^[^ ^30^ ^]^ or a quantum Hall effect,^[^ ^46^ ^]^ as have been previously shown in the LAO/STO systems. We anticipate that BSO‐based 2DEGs with even higher room‐temperature mobilities will be beneficial for transparent field‐effect transistor applications as well as a fundamental investigation of new physical phenomena. To this end lattice‐matched substrates for BSO interfacial 2DEG heterostructures will provide even more opportunities.

## 4 Experimental Section

### PLD Growth for BSO Pseudo‐Substrate

550 nm thick BSO buffer layers were grown on STO (001) substrates by pulsed‐laser deposition. Before deposition, STO substrates were treated by a buffered hydrofluoric acid etch and annealed in oxygen at 1000 °C for 6 h to create atomically smooth surfaces with single unit cell steps. The substrate was attached to a resistive heater and positioned ≈60 mm from the target. A KrF excimer laser (248 nm) was focused on a stochiometric BSO target to an energy density of 1.2 J cm^−2^ and pulsed at 5 Hz. BSO buffer layers were grown at substrate temperatures of 750 ℃ with an oxygen pressure of 120 mbar, and were slowly cooled down to room temperature under an oxygen pressure of 1 atm. After growth, the BSO film was leached by water for 15 s to create a SnO_2_‐termination and annealed in 1 atm of oxygen at 1150 ℃ for 2 h.

### MBE Growth of BSO and LSO Film

BSO and LSO thin films were grown in a Veeco GEN10 MBE system. Separate effusion cells containing barium (99.99% purity, Sigma‐Aldrich), SnO_2_ (99.996% purity, Alfa Aesar), lanthanum (99.996% purity, Ames Lab), and scandium (99.9% purity, Alfa Aesar) were heated. The fluxes of the resulting molecular‐beams emanating from the effusion cells were measured by a quartz crystal microbalance (QCM) before growth. A commercial ozone generator was used to produce the oxidant molecular beam source (≈10% ozone + 90% oxygen). The BSO film was grown in an adsorption‐controlled regime by supplying an excess SnO*_x_*‐flux.^[^ ^19^ ^]^ The background pressure of the oxidant, 10% O_3_ + 90% O_2_, was held at a constant ion gauge pressure of 1.0 × 10^−6^ Torr. Subsequently, LSO was grown on top of the BSO film using a layer‐by‐layer growth method. The fluxes of the La and Sc molecular beams were roughly calibrated by a QCM and then more precisely calibrated by growing La_2_O_3_ and Sc_2_O_3_ binary oxide films and measuring the growth rate using both X‐ray reflectivity and in situ reflection high‐energy electron diffraction (RHEED) oscillations.^[^ ^47^ ^]^ For the growth of both the BSO and LSO layers the substrate temperature was maintained between 830 and 850 °C, as measured by an optical pyrometer operating at a wavelength of 1550 nm.

### Electrical Transport Measurement

Transport measurements used four indium contacts in a van der Pauw geometry in a Quantum Design PPMS between 100 and 400 K. All resistance measurements were performed by sourcing an alternating dc current and measuring voltages at positive and negative current values. Hall measurements were performed by sweeping a magnetic field over a range from −20 to 20 kOe. The equations *n*_2D_ = *I*/[(*dV*_H_/*dB*)*q*] and *µ* = 1/(*n*_2D_ *qr*) were used to calculate 2D carrier density and mobility, where *Ι* is the dc current sourced, *V*_H_ is the Hall voltage, *q* is the electron charge, and *r* is the sheet resistance. The 3D carrier density was computed using the 2D carrier density and the thickness at which the electron density is zero as determined from the electron holopgraphy profile.

### STEM and EDS Measurement

The cross‐sectional sample for (S)TEM measurements was prepared via Ga^+^ ion beam milling at an accelerating voltage from 30 kV down to 5 kV using a dual‐beam focused ion beam system (FIB, Helios 450F1, Thermo Fisher Scientific). An aberration‐corrected TEM (JEM‐ARM300CF, JEOL) equipped with an energy dispersive x‐ray detector was used for TEM based measurements at 300 keV. The convergence semi‐angle of 23 mrad, and the collection angle ranges of 68–280 mrad, were set for HAADF image and EDS data, respectively. During acquisition of the EDS signal, the specimen drift was corrected during observation. Each elemental map is constructed by integrating the signal from Ba‐L, Sn‐L, La‐L, and Sc‐K characteristic x‐rays, respectively.

### Inline Holography Measurement

For inline electron holography, a through‐focal series of TEM bright‐field images was acquired using a 2 k × 2 k CCD camera (UltraScanXP 100FT, Gatan Inc.), varying the defocus values from −1800 to 1800 nm in 600 nm steps. An energy filter (Quantum spectrometer ER965, Gatan Inc.) was used to remove inelastically scattered electrons outside an energy window of 0 ± 5 eV. An objective aperture with a diameter of 20 µm was used to select the transmitted beam for bright‐field imaging. To minimize the effects of dynamical diffraction, all images were taken at diffraction condition off the $\left[1\bar{1}0\right]$ zone axis with the interface being kept in an edge‐on projection.^[^ ^48^ ^]^ The obtained inline electron holograms were used to reconstruct the phase shift of the transmitted beam using the full resolution wave reconstruction (FRWR) algorithm.^[^ ^49^ ^]^ The reconstructed phase images were converted into the map of the projected electrostatic potential by assuming the phase‐object approximation for a non‐magnetic material. The charge‐density map was obtained from the potential data using Poisson's equation.

## Conflict of Interest

The authors declare no conflict of interest.

## Supporting information

Supporting Information

## Data Availability

The data that support the findings of this study are available from the corresponding author upon reasonable request. Additional data related to the growth and structural characterization is available at https://doi.org/10.34863/tsb1-7w62.

## References

[1] A. Brinkman , M. Huijben , M. van Zalk , U. Zeitler , J. C. Maan , W. G. van der Wiel , G. Rijnders , D. H. A. Blank , H. Hilgenkamp , Nat. Mater. 2007, 6, 493. 17546035 10.1038/nmat1931

[2] N. Reyren , S. Thiel , A. D. Caviglia , L. Fitting Kourkoutis , G. Hammerl , C. Richter , C. W. Schneider , T. Kopp , A.‐S. Rüetschi , D. Jaccard , M. Gabay , D. A. Muller , J.‐M. Triscone , J. Mannhart , Science 2007, 317, 1196. 17673621 10.1126/science.1146006

[3] J. A. Bert , B. Kalisky , C. Bell , M. Kim , Y. Hikita , H. Y. Hwang , K. A. Moler , Nat. Phys. 2011, 7, 767.

[4] S. Banerjee , E. Onur , R. Mohit , Nat. Phys. 2013, 9, 626.

[5] A. D. Caviglia , M. Gabay , S. Gariglio , N. Reyren , C. Cancellieri , J. ‐M. Triscone , Phys. Rev. Lett. 2010, 104, 126803. 20366557 10.1103/PhysRevLett.104.126803

[6] M. Briggeman , M. Tomczyk , B. Tian , H. Lee , J. ‐W. Lee , Y. He , A. Tylan‐Tyler , M. Huang , C.‐B. Eom , D. Pekker , R. S. K. Mong , P. Irvin , J. Levy , Science 2020, 367, 769. 32054758 10.1126/science.aat6467

[7] F. Trier , G. E. D. K. Prawiroatmodjo , Z. Zhong , D. V. Christensen , M. v. Soosten , A. Bhowmik , J. M. G. Lastra , Y. Chen , T. S. Jespersen , N. Pryds , Phys. Rev. Lett. 2016, 117, 096804. 27610874 10.1103/PhysRevLett.117.096804

[8] A. Ohtomo , H. Y. Hwang , Nature 2004, 427, 423. 14749825 10.1038/nature02308

[9] C. He , T. D. Sanders , M. T. Gray , F. J. Wong , V. V. Mehta , Y. Suzuki , Phys. Rev. B 2012, 86, 081401(R).

[10] A. Annadi , A. Putra , Z. Q. Liu , X. Wang , K. Gopinadhan , Z. Huang , S. Dhar , T. Venkatesan , Ariando , Phys. Rev. B 2012, 86, 085450. 10.1103/PhysRevLett.107.146802 22112172

[11] Y. Z. Chen , N. Bovet , F. Trier , D. V. Christensen , F. M. Qu , N. H. Andersen , T. Kasama , W. Zhang , R. Giraud , J. Dufouleur , T. S. Jespersen , J. R. Sun , A. Smith , J. Nygard , L. Lu , B. Buchner , B. G. Shen , S. Linderoth , N. Pryds , Nat. Commun. 2013, 4, 1371. 23340411 10.1038/ncomms2394

[12] B. Himmetoglu , A. Janotti , H. Peelaers , A. Alkauskas , C. G. Van de Walle , Phys. Rev. B 2014, 90, 241204.

[13] T. R. Paudel , E. Y. Tsymbal , Phys. Rev. B 2017, 96, 245423.

[14] L. Bjaalie , B. Himmetoglu , L. Weston , A. Janotti , C. G. Van de Walle , New J. Phys. 2014, 16, 025005.

[15] X. Fan , W. Zheng , X. Chen , D. J. Singh , PLoS One 2014, 9, e91423. 24626191 10.1371/journal.pone.0091423 PMC3953397

[16] H. J. Kim , U. Kim , T. H. Kim , J. Kim , H. M. Kim , B.‐G. Jeon , W.‐J. Lee , H. S. Mun , K. T. Hong , J. Yu , K. Char , K. H. Kim , Phys. Rev. B 2012, 86, 165205.

[17] H. J. Kim , J. Kim , T. H. Kim , W.‐J. Lee , B.‐G. Jeon , J.‐Y. Park , W. S. Choi , D. W. Jeong , S. H. Lee , J. Yu , T. W. Noh , K. H. Kim , Phys. Rev. B 2013, 88, 125204.

[18] W.‐J. Lee , H. J. Kim , J. Kang , D. H. Jang , T. H. Kim , J. H. Lee , K. H. Kim , Annu. Rev. Mater. Res. 2017, 47, 391.

[19] H. Paik , Z. Chen , E. Lochocki , A. Seidner H , A. Verma , N. Tanen , J. Park , M. Uchida , S. Shang , B.‐C. Zhou , M. Brützam , R. Uecker , Z.‐K. Liu , D. Jena , K. M. Shen , D. A. Muller , D. G. Schlom , APL Mater. 2017, 5, 116107.

[20] A. Prakash , P. Xu , A. Faghaninia , S. Shukla , J. W. Ager 3rd , C. S. Lo , B. Jalan , Nat. Commun. 2017, 8, 15167. 28474675 10.1038/ncomms15167 PMC5424175

[21] A. P. Nono Tchiomo , W. Braun , B. P. Doyle , W. Sigle , P. van Aken , J. Mannhart , P. Ngabonziza , APL Mater. 2019, 7, 041119.

[22] J. Shiogai , K. Nishihara , K. Sato , A. Tsukazaki , AIP Adv. 2016, 6, 065305.

[23] U. Kim , C. Park , Y. M. Kim , J. Shin , K. Char , APL Mater. 2016, 4, 071102.

[24] J. Shin , Y. M. Kim , C. Park , K. Char , Phys. Rev. Appl. 2020, 13, 064066.

[25] Y. M. Kim , T. Markurt , Y. Kim , M. Zupancic , J. Shin , M. Albrecht , K. Char , Sci. Rep. 2019, 9, 16202. 31700133 10.1038/s41598-019-52772-8 PMC6838460

[26] A. Prakash , N. F. Quackenbush , H. Yun , J. Held , T. Wang , T. Truttmann , J. M. Ablett , C. Weiland , T. L. Lee , J. C. Woicik , K. A. Mkhoyan , B. Jalan , Nano Lett. 2019, 19, 8920. 31702928 10.1021/acs.nanolett.9b03825

[27] W.‐J. Lee , H. Lee , K.‐T. Ko , J. Kang , H. J. Kim , T. Lee , J.‐H. Park , K. H. Kim , Appl. Phys. Lett. 2017, 111, 231604.

[28] J. W. Park , D. F. Bogorin , C. Cen , D. A. Felker , Y. Zhang , C. T. Nelson , C. W. Bark , C. M. Folkman , X. Q. Pan , M. S. Rzchowski , J. Levy , C. B. Eom , Nat. Commun. 2010, 1, 94. 20981022 10.1038/ncomms1096

[29] H. J. Kim , U. Kim , H. M. Kim , T. H. Kim , H. S. Mun , B.‐G. Jeon , K. T. Hong , W.‐J. Lee , C. Ju , K. H. Kim , K. Char , Appl. Phys. Express 2012, 5, 061102.

[30] H. Lee , N. Campbell , J. Lee , T. J. Asel , T. R. Paudel , H. Zhou , J. W. Lee , B. Noesges , J. Seo , B. Park , L. J. Brillson , S. H. Oh , E. Y. Tsymbal , M. S. Rzchowski , C. B. Eom , Nat. Mater. 2018, 17, 231. 29403056 10.1038/s41563-017-0002-4

[31] E. Lesne , N. Reyren , D. Doennig , R. Mattana , H. Jaffres , V. Cros , F. Petroff , F. Choueikani , P. Ohresser , R. Pentcheva , A. Barhelemy , M. Bibes , Nat. Commun. 2014, 5, 4291. 25000146 10.1038/ncomms5291

[32] K. Song , S. Ryu , H. Lee , T. R. Paudel , C. T. Koch , B. Park , J. K. Lee , S. Y. Choi , Y. M. Kim , J. C. Kim , H. Y. Jeong , M. S. Rzchowski , E. Y. Tsymbal , C. ‐B. Eom , S. H. Oh , Nat. Nanotechnol. 2018, 13, 198. 29402977 10.1038/s41565-017-0040-8

[33] P. Singh , B. J. Brandenburg , C. P. Sebastian , P. Singh , S. Singh , D. Kumar , O. Parkash , Jpn. J. Appl. Phys. 2008, 47, 3540.

[34] B. K. Choudhury , K. V. Rao , R. N. P. Choudhury , J. Mater. Sci. 1989, 24, 3469.

[35] K. Song , T. Min , J. Seo , S. Ryu , H. Lee , Z. Wang , S. Y. Choi , J. Lee , C. B. Eom , S. H. Oh , Adv. Sci. 2021, 8, 2002073. 10.1002/advs.202002073 PMC8292910 34029001

[36] D. Yoon , S. Yu , J. Son , NPG Asia Mater. 2018, 10, 363.

[37] H. Yun , A. Prakash , T. Birol , B. Jalan , K. A. Mkhoyan , Nano Lett. 2021, 21, 4357. 33973791 10.1021/acs.nanolett.1c00966

[38] A. Lubk , M. D. Rossell , J. Seidel , Y. H. Chu , R. Ramesh , M. J. Hytch , E. Snoeck , Nano Lett. 2013, 13, 1410. 23418908 10.1021/nl304229k

[39] D. Marrocchelli , L. Sun , B. Yildiz , J. Am. Chem. Soc. 2015, 137, 4735. 25751017 10.1021/ja513176u

[40] P. Hirel , P. Carrez , E. Clouet , P. Cordier , Acta Mater. 2016, 106, 313.

[41] H. M. Ng , D. Doppalapudi , T. D. Moustakas , N. G. Weimann , L. F. Eastman , Appl. Phys. Lett. 1998, 73, 821.

[42] N. Miller , E. E. Haller , G. Koblmüller , C. Gallinat , J. S. Speck , W. J. Schaff , M. E. Hawkridge , K. M. Yu , J. W. Ager , Phys. Rev. B 2011, 84, 075315.

[43] D. H. Jang , W.‐J. Lee , E. Sohn , H. J. Kim , D. Seo , J.‐Y. Park , E. J. Choi , K. H. Kim , J. Appl. Phys. 2017, 121, 125109.

[44] C. Guguschev , D. Klimm , M. Brützam , T. M. Gesing , M. Gogolin , H. Paik , A. Dittmar , V. J. Fratello , D. G. Schlom , J. Cryst. Growth 2019, 528, 125263.

[45] Z. Galazka , K. Irmscher , S. Ganschow , M. Zupancic , W. Aggoune , C. Draxl , M. Albrecht , D. Klimm , A. Kwasniewski , T. Schulz , M. Pietsch , A. Dittmar , R. Grueneberg , U. Juda , R. Schewski , S. Bergmann , H. Cho , K. Char , T. Schroeder , M. Bickermann , Phys. Status Solidi 2021, 218, 2100016.

[46] Y. Z. Chen , F. Trier , T. Wijnands , R. J. Green , N. Gauquelin , R. Egoavil , D. V. Christensen , G. Koster , M. Huijben , N. Bovet , S. Macke , F. He , R. Sutarto , N. H. Andersen , J. A. Sulpizio , M. Honig , G. E. Prawiroatmodjo , T. S. Jespersen , S. Linderoth , S. Ilani , J. Verbeeck , G. Van Tendeloo , G. Rijnders , G. A. Sawatzky , N. Pryds , Nat. Mater. 2015, 14, 801. 26030303 10.1038/nmat4303

[47] J. Sun , C. T. Parzyck , J. H. Lee , C. M. Brooks , L. F. Kourkoutis , X. Ke , R. Misra , J. Schubert , F. V. Hensling , M. R. Barone , Z. Wang , M. E. Holtz , N. J. Schreiber , H. Paik , T. Heeg , P. Schiffer , D. A. Muller , K. M. Shen , D. G. Schlom , Phys. Rev. Mater. unpublished.

[48] P. Formanek , E. Bugiel , Ultramicroscopy 2006, 106, 292. 16330148 10.1016/j.ultramic.2005.09.003

[49] C. T. Koch , Micron 2014, 63, 69. 24239416 10.1016/j.micron.2013.10.009

