# Supplementary material for: Oxide Two‐Dimensional Electron Gas with High Mobility at Room‐Temperature
Source: Adv Sci (Weinh). 2022 Feb 20;9(12):2105652. doi: 10.1002/advs.202105652 (PMC9036036; doi:10.1002/advs.202105652)
Supplement: Supplementary file 1 — Supporting Information [file ADVS-9-2105652-s001.pdf]

ADVANCED SCIENCE
Open Access

Supporting Information

for *Adv. Sci.*, DOI 10.1002/advs.202105652

Oxide Two-Dimensional Electron Gas with High Mobility at Room-Temperature

*Kitae Eom, Hanjong Paik, Jinsol Seo, Neil Campbell, Evgeny Y. Tsymbal, Sang Ho Oh, Mark S. Rzchowski, Darrell G. Schlom and Chang-Beom Eom**

Supporting Information for

Oxide two-dimensional electron gas with high mobility at room-temperature

Kitae Eom, Hanjong Paik, Jinsol Seo, Neil Campbell, Sang Ho Oh, Mark Rzchowski, Darrell G. Schlom, and Chang-Beom Eom*

Contents

Section S1. The effect of post treatment on the BSO pseudo-substrate
Figure S1. Structural characterization of PLD-grown BSO thin films before and after thermal treatment.

Section S2. MBE growth of BSO films
Figure S2. RHEED intensity oscillations during the MBE growth of BSO films

Section S3. Dislocation density analysis
Figure S3. A magnified STEM-HAADF image near the LSO/BSO interface regions along the [110] zone axis.
Figure S4. Dislocation density calculations using weak beam dark field images
Figure S5. STEM-HAADF images and geometric phase analysis of the BSO/STO interface

Section S4. Critical thickness threshold for conductivity at the LSO/BSO interface
Figure S6. Thickness-dependent evolution of *in-situ* RHEED intensity oscillations during the PLD growth of LSO films.
Figure S7. Room temperature sheet conductivity as a function of the number of LSO unit cells.

Section S5. Inline holography measurement
Figure S8. Charge distribution in the LSO (4 u.c.)/BSO interface
Figure S9. Atomic-column resolved STEM-EDS composition profiles

Section S6. Possible pathway to achieve high RT mobility without ex-situ thermal treatment

*Correspondence should be sent to eom@engr.wisc.edu.

Section S1. Structural characterization of PLD-grown BSO thin films before and after thermal treatment. The effect of post treatment on the BSO pseudo-substrate.

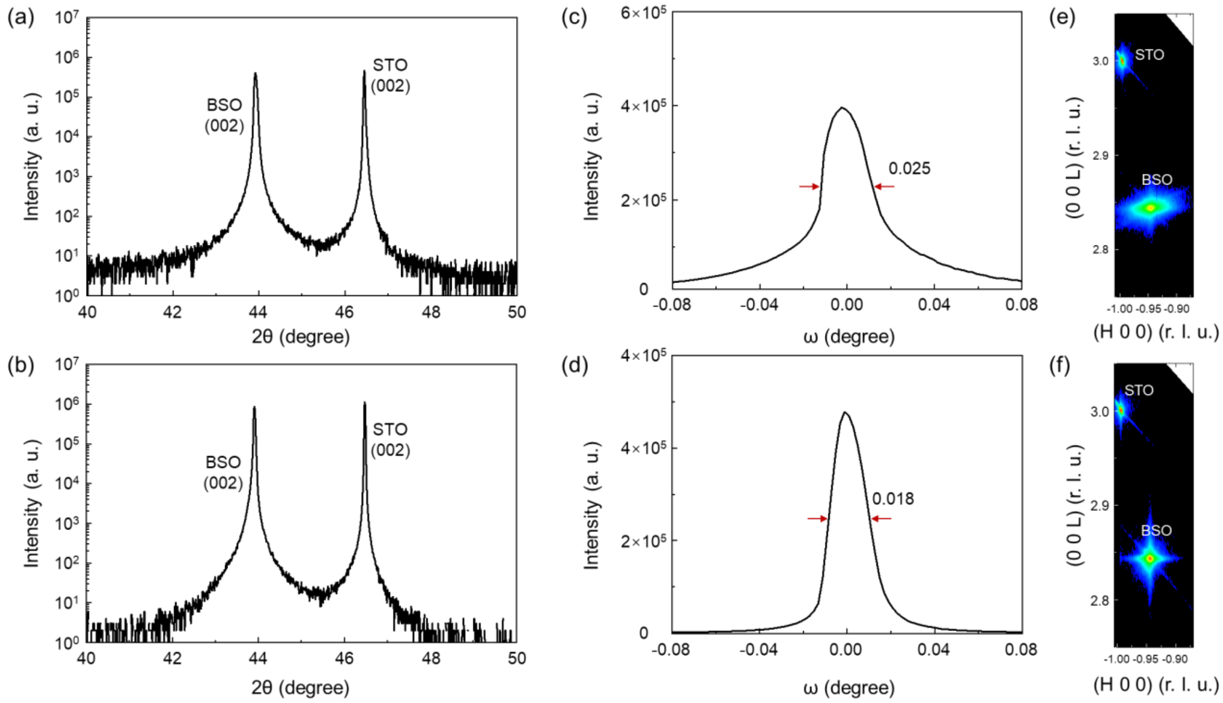

Figure S1. Structural characterization of PLD-grown BSO thin films before and after thermal treatment. θ-2θ scans (a) before and (b) after post treatment. Rocking curves of the BSO 002 peak (c) before and (d) after post treatment. The changes of the full width at half max (FWHM) of the BSO 002 and corresponding RSM result taken around the STO 103 reflection (e) before and (f) after post treatment reflects the dislocation annihilation effect.

Section S2. MBE growth of BSO films

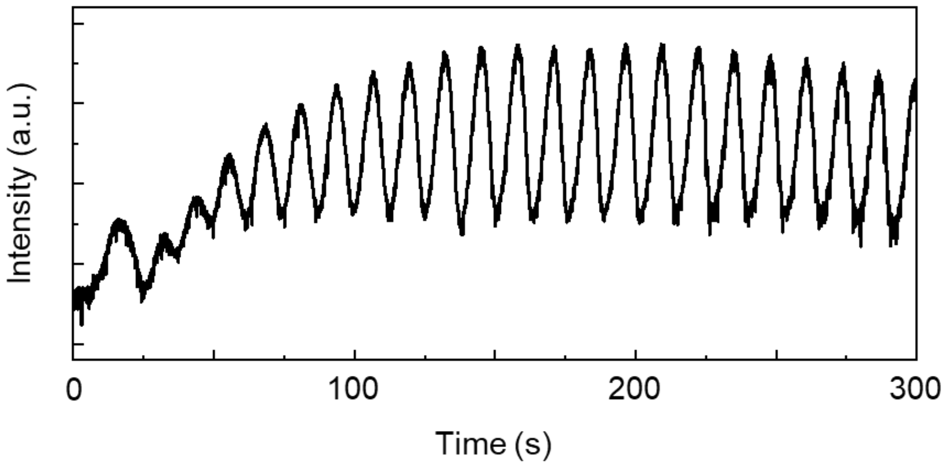

Figure S2. RHEED intensity oscillations during the MBE growth of BSO films

Section S3. Dislocation density analysis

The threading dislocation density was measured by counting dislocation lines in TEM weak beam dark field images following the equation below,

$$\rho = \frac{N}{Lt} \tag{0.1}$$

where N is the total number of dislocations in a field of view, L is the total length of the field of view and t is the thickness of the TEM specimen. The weak beam dark field images were taken by selecting the in-plane 002 beam. The total field of view, L of LSO (10 u.c.)/BSO (45 nm)/BSO (550 nm) and LSO (10 u.c.)/BSO (60 nm) specimens were 3.629 μm and 3.057 μm, respectively. To measure the total number of dislocations, N, a few lines were drawn in the in-plane direction, and the number of intersections with the dislocations was counted and averaged. Considering that the TEM specimen is wedge-shaped and that threading dislocations can escape through the surface of a thin TEM specimen, the number of representative dislocations was counted near the bottom interfaces (BSO/STO interface). The number of dislocations counted are 128 and 332 for LSO (10 u.c.)/BSO (45 nm)/BSO (550 nm) and LSO (10 u.c.)/BSO (90 nm) specimens, respectively. The thickness of specimens, t determined by using the EELS log-ratio method,[1] were 110.67 nm and 98.87 nm on average with small thickness gradients for the LSO (10 u.c.)/BSO (45 nm)/BSO (550 nm) and LSO (10 u.c.)/BSO (60 nm) specimens, respectively.

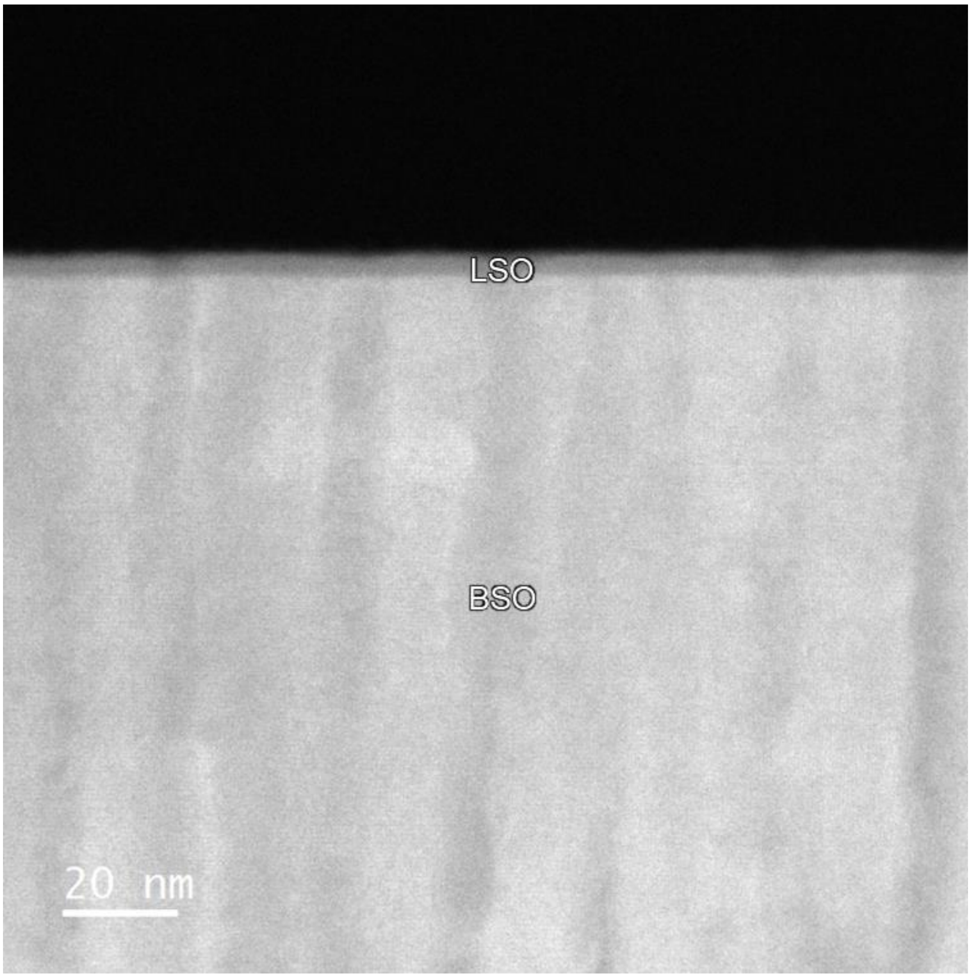

Figure S3. A magnified STEM-HAADF image near the LSO/BSO interface regions with a zone axis of [110]. Threading dislocations propagate in the film growth direction from the BSO/STO interface into the LSO layer.

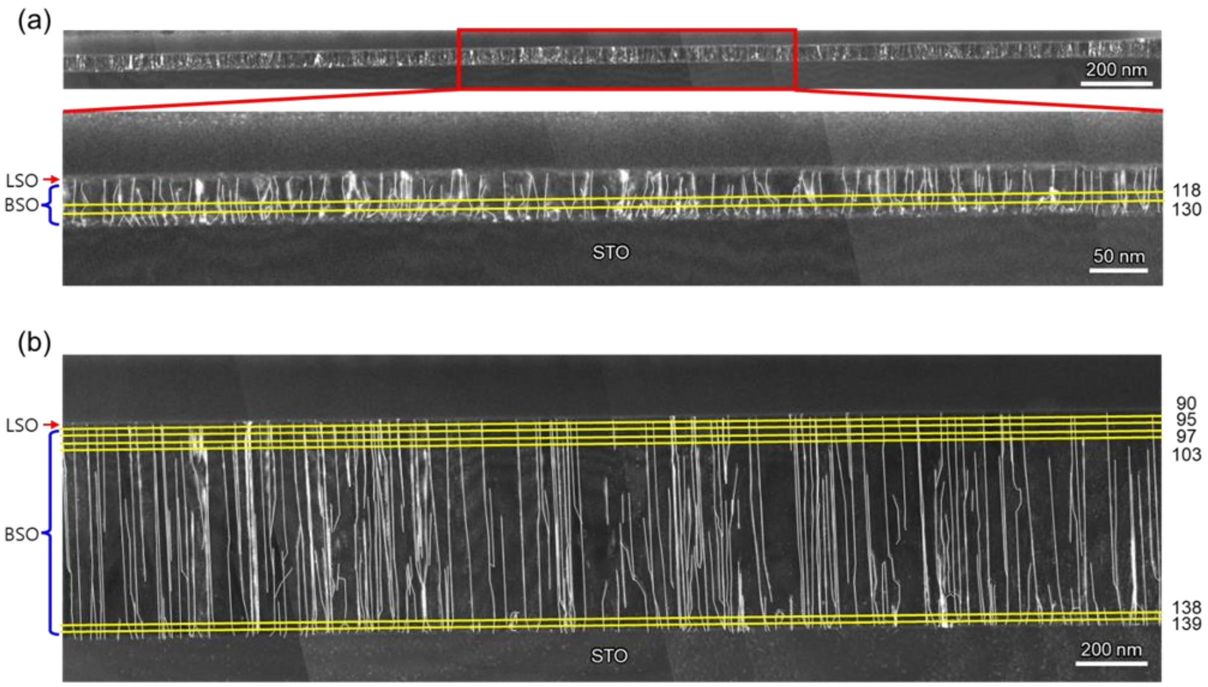

Figure S4. Dislocation density calculations made by using weak beam dark field images from (a) LSO (10 u.c.) / BSO (60 nm) / STO without the BSO pseudo-substrate and (b) LSO (10 u.c.) / BSO (45 nm) grown on the BSO pseudo-substrate. An in-plane 200 beam was excited for imaging. Several images are stitched together for both samples due to the limited field of view. Dislocations are highlighted with white lines. To extract dislocation densities, we drew lines parallel to the film (yellow lines). The intersections were counted, and averaged numbers were used to estimate the dislocation density. The total fields of view are 3.1 μm and 3.6 μm for LSO (10 u.c.) / BSO (60 nm) / STO and LSO (10 u.c.) / BSO (45 nm) grown on a BSO psuedo-substrate, respectively.

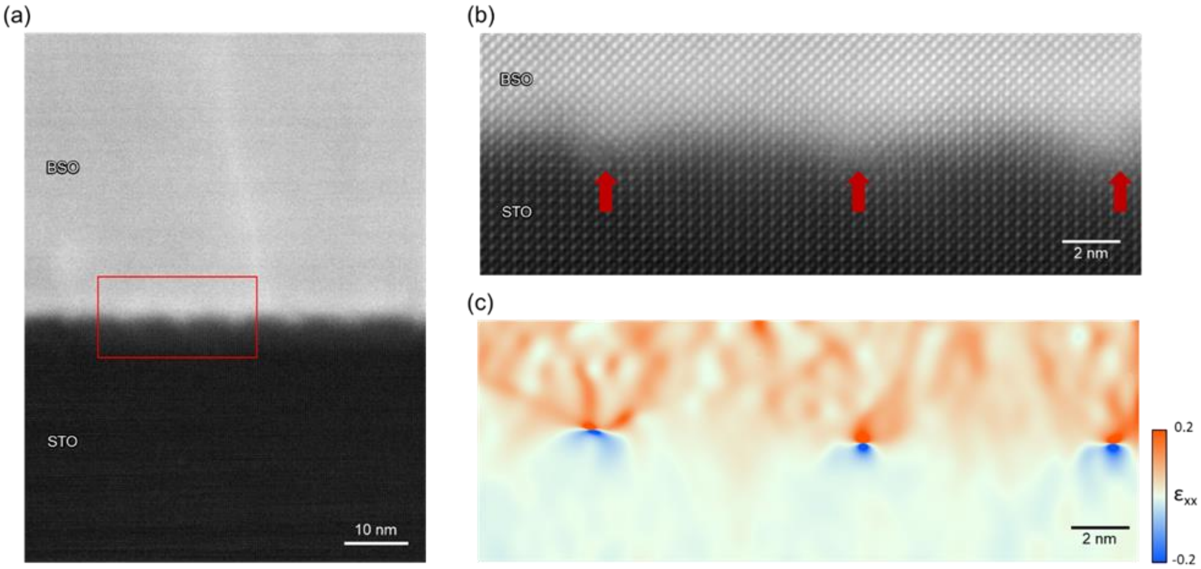

Figure S5. STEM-HAADF images and geometric phase analysis on the BSO/STO interface at the LSO (10 u.c.)/BSO (45 nm) grown on a BSO pseudo-substrate. (a) STEM HAADF image with low magnification. (b) atomic-column resolved STEM-HAADF image from the area marked with the red rectangle in (a). The red arrows indicate misfit dislocations. (c) In-plane strain (ε_{xx}) map from GPA analysis, which is obtained in the same area as (b).

Section S4. Critical thickness threshold for conductivity at the LSO/BSO interface

To investigate the critical thickness threshold for conductivity at the LSO/BSO interface, LSO films were grown by PLD on MBE-grown 90 nm thick BSO/STO films. The LSO target was ablated using a KrF (248 nm) excimer laser at a repetition rate of 3 Hz and with a fluence of 1.8 J/cm^2. The substrate to target distance was 62 mm. The LSO films were grown at a temperature of 750 ℃ with an oxygen pressure of 10 mbar, and were slowly cooled down to room temperature under an oxygen pressure of 1 atm. Before the growth, the BSO/STO film was leached by DI water for 15 sec to obtain an SnO_2-terminated surface.[2] The LSO film thickness is controlled by monitoring the RHEED oscillations (Figure S6).

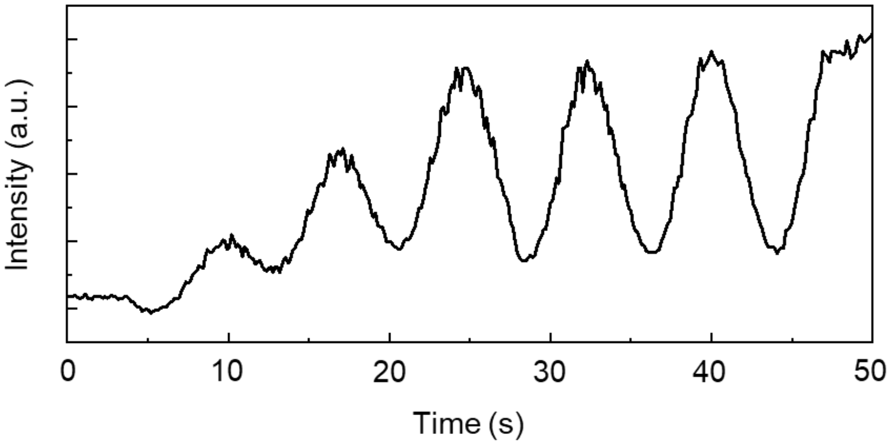

Figure S6. Thickness-dependent evolution of *in-situ* RHEED intensity oscillations during the PLD growth of LSO films.

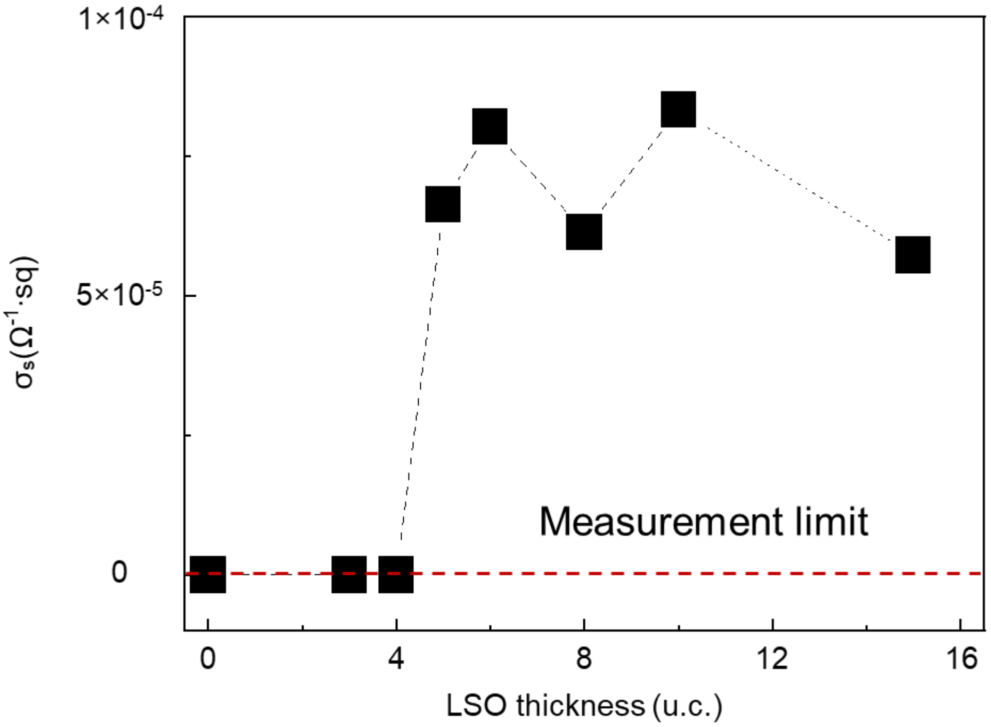

Figure S7. Sheet conductivity at room temperature as a function of the number of LSO unit cells. LSO layers were grown by PLD on 90 nm thick BSO / STO (001) samples that had been grown by MBE and leached by water to make SnO_2-terminated surfaces. This plot indicates that a conducting interface is formed when the thickness of the LSO layer is more than 4 u.c., clearly showing the LSO critical thickness limit for forming a conducting 2DEG interface on BSO.

Section 5. Inline holography measurement

In our previous report, it has been demonstrated that the inline holography technique is capable of measuring highly confined electron 2DEGs even at interfaces having a low spatial frequency issue.[3] The charge density map/profile should be carefully considered because of the mean inner potential and a limited spatial resolution of the phase image. The estimated mean inner potentials of LSO and BSO are 17.1 and 16.3, respectively,[4] which may cause a small artificial peak at the interface in the charge density maps and profiles (Figure 5b and Figure S8). Nevertheless, it is still reasonable to conclude distinctly different charge confinement at the LSO (10 u.c.) / BSO (90 nm) interface compared to that of the LSO (4 u.c.) / BSO (90 nm) interface.
One way to quantify the impact of defects on carrier localization is to compare the room-temperature Hall carrier density with the room-temperature in-line holography charge density. It should be noted that Hall Effect measurements are only sensitive to mobile carriers while holography is sensitive to the net charge density that includes mobile and localized carriers. We first note that comparison of the Hall 2D carrier density, 1.7×10^{13} cm^{-2}, with the holography 3D density, 5×10^{21} cm^{-3}, gives a much smaller conducting channel thickness (~0.03 nm) than the holography measurement (~3 nm). This is likely because the in-line holography technique usually over-estimates the charge carrier density [Nat. Nanotech. 13, 198 (2018)]. At the same time, this indicates that charge trapping by defects has a large influence on the conduction of our 2DEG.

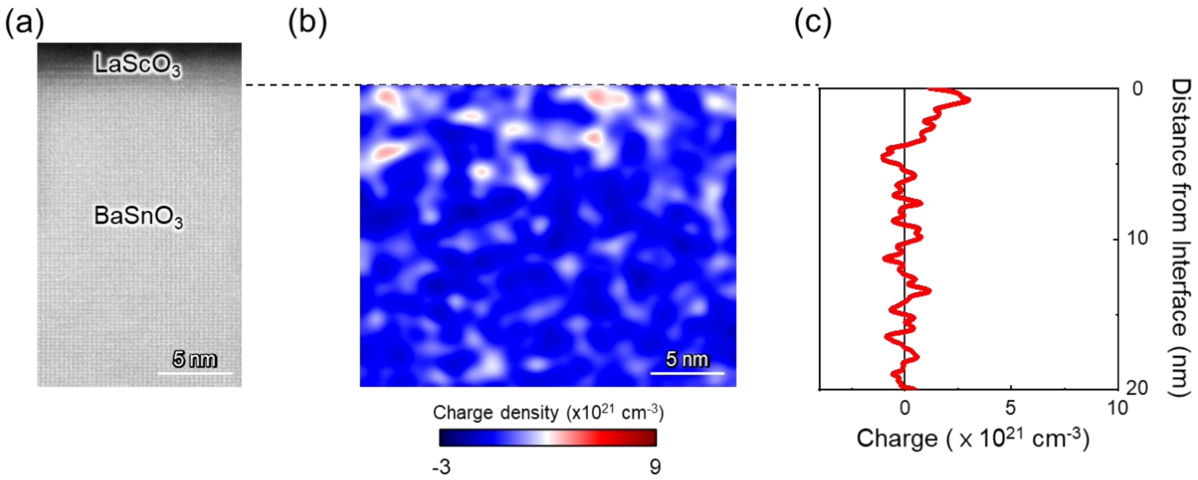

Figure S8. Charge distribution in the LSO (4 u.c.)/BSO interface. (a) STEM HAADF images are shown next to (b) the charge density maps and (c) 1D electron density profiles obtained by in-line electron holography for the LSO (4 u.c.) / BSO (90 nm) grown directly on an STO substrate.

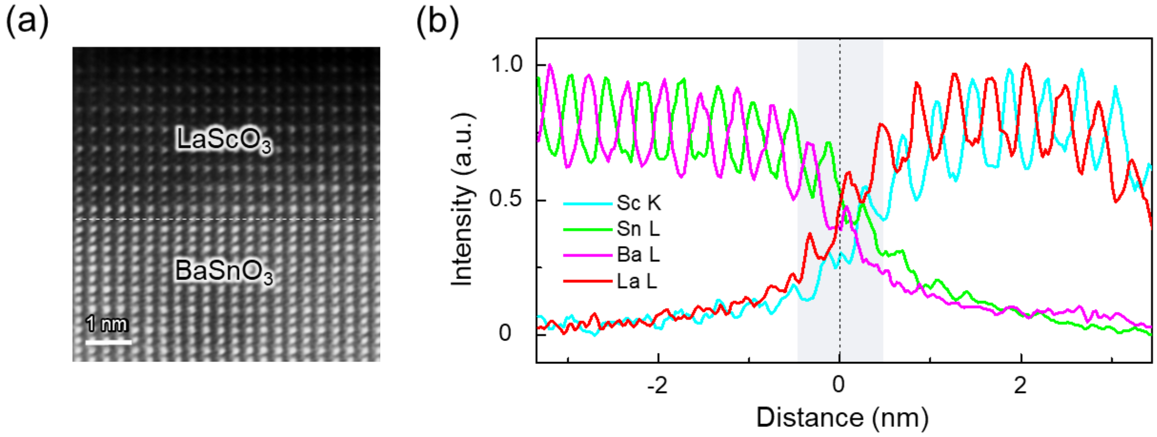

Figure S9. Atomic-column resolved STEM-EDS composition profiles. (a) Atomic resolution HAADF STEM image of the LSO (10 u.c.) / BSO (90 nm) / STO along the [001] zone axis of STO (b) normalized intensity profiles of STEM-EDS obtained at the LSO/BSO interface, which is constructed by selecting Sc-K, Sn-L, Ba-L and La-L characteristic X-rays, respectively.

Section 6. Possible pathway to achieve high RT mobility without ex-situ thermal treatment

A key limitation to realizing high mobility BSO-based 2DEGs is the lack of a suitable substrate. Our ex-situ high temperature treatment effectively reduces the dislocation density, but our BSO film still suffers from high concentrations of threading dislocations with a density near 4×10^{10} cm^{-2}. Suitable single-crystal BSO substrates would alleviate the ex-situ treatment.[6] Unfortunately, the irregular shapes, small size, and un-intentional doping in BSO single crystals make it difficult to use as a substrate for 2DEG study.

Recently, synthesis of large single crystals of two perovskite substrates that are well lattice matched to $BaSnO_3$ have been reported: $LaInO_3$ and the double-perovskite Ba_2ScNbO_6.[7] The excellent lattice matching of these new perovskite substrates to BSO, suggests possibilities for dislocation-free BSO thin films. Future work employing these well lattice-matched substrates may help to achieve high mobility BSO 2DEGs without an ex-situ treatment.

References

[1] K. Iakoubovskii, K. Mitsuishi, Y. Nakayama, K. Furuya, *Microscopy research and technique* **2008,** 71, (8), 626-631.

[2] W. -J. Lee, H. Lee, K. -T. Ko, J. Kang, H. J. Kim, T. Lee, J.-H. Park, K. H. Kim, *Appl. Phys. Lett.* **2017**, 111, 231604.

[3] J. Seo, C. T. Koch, S. Ryu, C.-B. Eom, S. H. Oh, *Ultramicroscopy* **2021**, 113236.

[4] L.-M. Peng, *Acta Crystallographica Section A: Foundations of Crystallography* **1998,** 54, 481-485.

[5] K. Song, S. Ryu, H. Lee, T. R. Paudel, C. T. Koch, B. Park, J. K. Lee, S. Y. Choi, Y. M. Kim, J. C. Kim, H. Y. Jeong, M. S. Rzchowski, E. Y. Tsymbal, C. -B. Eom, S. H. Oh, *Nat. Nanotechnol.* **2018**, 13, 198-203.

[6] Z. Galazka, R. Uecker, K. Irmscher, D. Klimm, R. Bertram, A. Kwasniewski, M Naumann, R. Schewski, M Pietsch, U. Juda, A Fiedler, M. Albrecht, S. Ganschow, T. Markurt, C Guguschev, M. Bickermann, *J. Phys.:Condens. Matter*. **2017**, 29, 075701.

[7] Z. Galazka, K. Irmscher, S. Ganschow, M. Zupancic, W. Aggoune, C. Draxl, M. Albrecht, D. Klimm, A. Kwasniewski, T. Schulz, M. Pietsch, A. Dittmar, R. Grueneberg, U. Juda, R. Schewski, S. Bergmann, H. Cho, K. Char, T. Schroeder, M. Bickermann, *Phys Status Solidi* **2021**, 218, 2100016.
